# Stepped Care for Maternal Mental Health: A Case Study of the Perinatal Mental Health Project in South Africa

**DOI:** 10.1371/journal.pmed.1001222

**Published:** 2012-05-29

**Authors:** Simone Honikman, Thandi van Heyningen, Sally Field, Emily Baron, Mark Tomlinson

**Affiliations:** 1 Perinatal Mental Health Project, The Alan J Flisher Centre for Public Mental Health, University of Cape Town, Western Cape, South Africa; 2 Department of Psychology, Stellenbosch University and The Alan J Flisher Centre for Public Mental Health, Stellenbosch University and University of Cape Town, Western Cape, South Africa

## Abstract

As one article in a series on Global Mental Health Practice, Simone Honikman and colleagues from South Africa provide a case study of the Perinatal Mental Health Project, which delivered mental health care to pregnant women in a collaborative, step-wise manner, making use of existing resources in primary care.

**Summary Points**

- Maternal mental health is largely neglected in low- and middle-income countries.
- There is no routine screening or treatment of maternal mental disorders in primary care settings in South Africa.
- The Perinatal Mental Health Project (PMHP) developed an intervention to deliver mental health care to pregnant women in a collaborative, step-wise manner making use of existing resources in primary care.
- Over a 3-year period, 90% of all women attending antenatal care in the maternity clinic were offered mental health screening with 95% uptake. Of those screened, 32% qualified for referral to counselling.
- Through routine screening and referral, the PMHP model demonstrates the feasibility and acceptability of a stepped care approach to provision of mental health care at the primary care level.

*This case study is part of the* PLoS Medicine *series on Global Mental Health Practice.*

## Maternal Mental Health in South Africa

Common mental disorders such as anxiety and depression are the third leading causes of disease burden globally for women between 14 and 44 years of age [1]. By 2030, these are expected to rise to first place, ranked above heart disease and road traffic injuries [2]. A recent systematic review reveals that maternal mental disorders are approximately three times more prevalent in low- and middle-income countries (LMICs) than in high-income countries (HICs), where the related burden of disease estimates range between 5.2% and 32.9% [3],^4^. In HICs, maternal suicide is the leading cause of death during the perinatal period, and while there is a relative dearth of information about maternal suicide in LMICs, the estimates are similarly high [5],[6]. Untreated maternal mental illness affects infant and child growth [7] and the quality of child care [8], resulting in compromised child development [4],[9].

Community-based epidemiological studies in South Africa have shown high prevalence rates of depressed mood amongst pregnant and postnatal women. In a low-income, informal settlement outside of Cape Town, 39% of pregnant women screened positive on the Edinburgh Postnatal Depression Scale (EPDS) for depressed mood [10] and 34.7% of postnatal women were diagnosed with depression [11]. In a rural area of KwaZulu-Natal province with high HIV prevalence, 47% of women were diagnosed with depression in their third trimester of pregnancy [12].

Despite high levels of antenatal and postnatal depression, there is no routine screening or treatment of maternal mental disorders in primary care settings in South Africa. Maternal care involves an average of three antenatal clinic visits with coverage of 92% of the pregnant population [13]. The antenatal care is predominantly focused on physical examination [14], whereas during the post-partum period, the health care focus is commonly on the infant for immunisation, growth monitoring, and HIV testing. The lack of integration between maternal health services, child health services, and mental health services in primary care creates a large gap in the screening and treatment of maternal mental disorders [15].

If women are referred for mental health services, they are often required to incur additional costs related to transport and child care, and loss of income to attend appointments. Women may also be referred to services at a different site, which exacerbates these costs and frequently results in poor uptake [16]. There is a lack of routine programmatic maternal mental health care in South Africa. This is despite evidence that such interventions may be successfully implemented in primary care settings [17],[18].

## Development of the Model

The Perinatal Mental Health Project (PMHP), based at the Mowbray Maternity Hospital in the Western Cape Province of South Africa, has developed a stepped care intervention for maternal mental health that is integrated into antenatal care. Mowbray Maternity Hospital is a secondary level maternity hospital, linked to the University of Cape Town, and located centrally within the city. The PMHP services are based at the hospital within the Midwife Obstetric Unit (MOU), which provides a primary level antenatal clinic. This unit serves women, with low obstetric risk, from the surrounding areas. The clinic sees approximately 150 women per month for their first antenatal “booking” visit, and there are approximately two midwives and one nursing assistant on daily duty.

Midwives at the MOU are trained to screen women routinely for maternal mood disorders during their antenatal visits. Those who screen positive are referred to on-site counsellors who also act as case managers. Where specialist intervention is indicated, women are referred to an on-site psychiatrist. The PMHP works directly with facility managers and health workers through collaborative partnerships, focusing on problem solving and capacity development in the primary health care system.

Before launching the service in 2002, the PMHP began a process of planning and design with practitioners working in the fields of social work, psychology, psychiatry, and midwifery. A draft service model was developed and this was presented for comment and permission to a range of stakeholders, including provincial representatives in the related sub-directorates of the Department of Health, senior and junior health managers and lead clinicians at the proposed service site, as well as all cadres of health and administrative staff working within the primary care obstetric facility.

Simultaneously, training and capacity-building workshops were conducted with midwives and nursing staff based at the hospital. From these it emerged that on-going mental health training for general health workers would form an integral part of the PMHP intervention.

## Implementation

### Training for Effective Task-Sharing

The Project includes training for sharing tasks, which employs an informal, participatory style of engagement with participants. On-going engagement with staff through training and supervision has assisted in motivating staff to engage with their own mental health needs, and has helped nursing staff to manage their workload more effectively and address feelings of “burn out.”

Many of the PMHP training components are available in manual format and include basic knowledge on maternal mental health, including epidemiological, social, clinical, and management issues; development of basic counselling skills; training in mental health screening procedures; and strategies for maximising the success of referrals.

### Stepped Care

All women are offered screening at their first antenatal visit by nurses and midwives, during routine history taking (see Table 1). The EPDS [19] is used, as it has been validated for use in South Africa [20], and a risk factor assessment (RFA) tool is used as well. The RFA was designed by the PMHP and is a yes/no tick-form consisting of 11 risk factors for mental distress. It is used to augment the sensitivity of the EPDS by taking into account the local context [21]–[23].

**Table 1:** PMHP stepped care model and organogram.

	Step	When	By Whom	With Whom	On-Going Processes^a^
1	**Stakeholder engagement**	At initial start-up of the project and then on an on-going basis	PMHP management and clinical team	Facility staff and management	Liaison with facility staff and management
2	**Training**	At start up and on-going as staff rotate through the antenatal clinic	PMHP Clinical Services Coordinator using PMHP training and manuals	Primary health care staff	Supervision and debriefing for staff
3	**Screening**	At women's first antenatal visit	Midwives and nurses using EPDS, PMHP RFA	Pregnant women	Referral to PMHP counselling team
4	**Counselling**	At subsequent antenatal visits, or at the convenience of the women	PMHP Clinical Coordinator, PMHP counsellors (lay and clinical psychologist)	Pregnant women	Referral to psychiatrist, social worker, or external resources (e.g., shelter, refugee centre); liaison with referring counsellor
5	**Psychiatry**	Scheduled appointments twice a month at the antenatal clinic	Psychiatrist	Pregnant women	Referral to social worker, or allied professionals
6	**Supervision**	Daily for screening staff, weekly for PMHP counselling staff, twice/month peer supervision for PMHP counsellors	Clinical Services Coordinator and peer supervision for counsellors	Screening staff, PMHP counselling staff	On-going monitoring and evaluation. Reporting to PMHP management team

a Strict monitoring and evaluation occurs at each step.

After signing informed consent, women self-administer the mental health screening questionnaires in private. In this urban setting, most women are literate, but midwives assist those who experience difficulties with the questionnaires. All forms are available in English, Afrikaans, isiXhosa (local languages), and French. The latter is provided to Francophone refugee women who attend the facility.

Screening scores are calculated by the midwives and screening data is recorded for monitoring. An encoded score is entered in the women's clinic records, as well as the relevant action arising from the screening. Women who meet a cut-off of 13 and above on the EPDS and/or 3 and above on the RFA are referred for on-site counselling. Counselling appointments are made to coincide with subsequent antenatal visits or when convenient for the women.

Individual counselling is provided free of charge, on an appointment basis, for up to 1 year post-partum. A full-time clinical psychologist co-ordinates the clinical services. She provides a liaison role with personnel at the hospital, counsels clients, and manages the counselling team. The team includes a lay counsellor and another psychologist, as well as a psychiatrist who works on a part-time basis. A number of therapeutic modalities are used in the counselling sessions, including psycho-education, bereavement counselling, problem solving, and interpersonal therapy. Alcohol and substance use, which is ascertained by nurses as a part of routine history-taking, is explored further in counselling. Women who present with alcohol and substance abuse are referred to the hospital social worker for further intervention, according to the management protocol of the maternity facility.

Each counsellor manages her own case load and occasionally collaborates with psychiatrists and allied health workers. Care is frequently supplemented by liaison with external agencies such as non-governmental organisations. Furthermore, women receive consistent and structured follow-up management including telephonic contact for those who are unable to attend the facility or who default counselling appointments.

The psychiatrist provides a fortnightly session at the obstetric facility. She liaises with the referring counsellor, who retains the central role in therapeutic management of the client. Women who are considered by counsellors to require psychiatric consultation are first engaged in a process to explore whether they would in fact take up psychiatric services. This enables counsellors to rationalise referrals to psychiatry for those clients who are most likely to engage positively with this care.

Every woman counselled receives a routine six-week postnatal follow up phone call. This takes the form of a feedback questionnaire that is designed to elicit information about the birthing experience, adjustment to life with the baby, the experience of counselling, and whether further intervention or referral to external resources is required. This contact often takes the form of a telephonic counselling session. This may be useful where women are unable to access the service, but may still require follow-up care.

Regular clinical supervision of counselling staff is provided through peer support protocols, and an external clinical supervisor. The clinical services co-ordinator is involved in regular supervision of the nursing staff. A structured and consistent audit of screening and clinical services is conducted on a monthly basis. Data from the screening and service delivery are collated and reported to clinical management staff in the maternity facility and to the PMHP team.

## Impact

**Figure 1:**
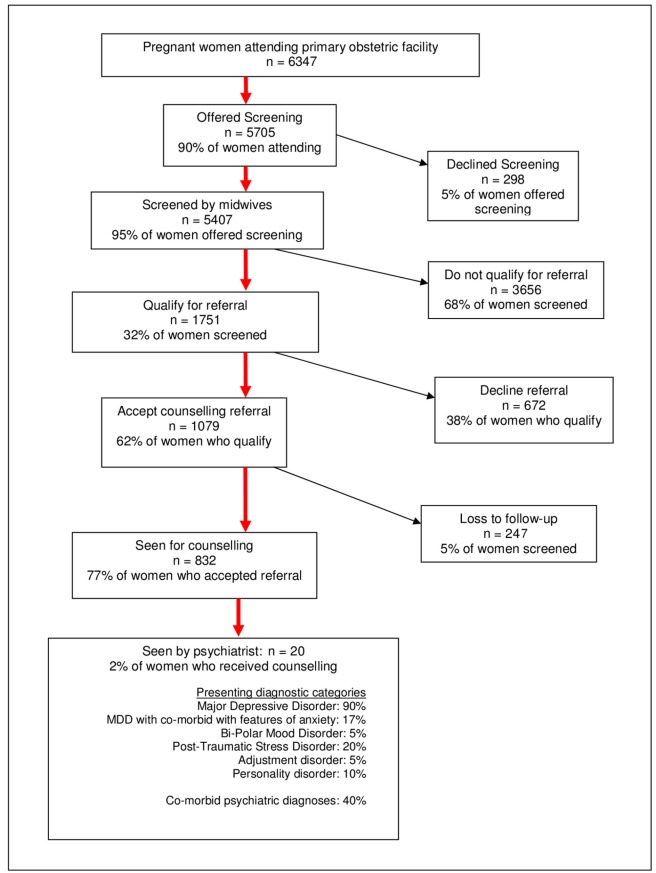
Screening coverage and pattern of service use from 01 July 2008–30 June 2011.

From July 2008 to the end of June 2011, 90% of 6,347 women who attended the facility for primary level care were offered mental health screening (see Figure 1). During this time, 95% of women accepted the screening. The mean age of women screened was 25 years, with the mean gestation at screening being 24 weeks. A large proportion of women (47.1%) were in their first pregnancy.

Of the 5,407 screened, 32% qualified for referral to a counsellor, and 62% (1,079 women) of those who qualified agreed to be referred. Of this latter group, 77% attended their appointments and received an average of 2.7 face-to-face sessions. A total of 1,981 counselling sessions were conducted, of which 832 were first sessions. Reasons cited by women for missed or cancelled counselling appointments related partly to logistical or personal resource issues and partly to their perception that their problems had improved.

A small proportion (2%, *n* = 20) of the women who were counselled were referred and seen by the PMHP psychiatrist. Of these women, 75% reported inadequate partner or family support, 45% reported past psychiatric problems, 40% reported past or present abuse (any form), and 5% had problems with substance abuse. The diagnoses for these women, made by the psychiatrist, appear in Figure 1.

From the beginning of 2010, formal postnatal evaluation procedures were instituted. Counsellors attempted telephone contact with all counselled women. For the 12-month period ending 30 June 2011, 170 postnatal follow-up phone calls were made. A preliminary analysis of women's self-reported data reveals that at 6–10 weeks post-partum, 87.8% of women reported an improvement in their presenting problem, 79.9% of mothers reported to be coping at the time of the telephone assessment, and 74.6% reported positive mood at the time of the assessment. Furthermore, 91.7% of these women rated the sessions as a positive experience.

## Barriers and Facilitators

In low-resource primary care settings, where common mental disorders are often overlooked, integrating screening into routine antenatal procedures, such as we observed with the PMHP, has the potential to narrow the treatment gap significantly. Universal screening allows for early detection of psychological distress in most cases [23]. Women who meet the criteria may then be immediately referred to counsellors and the need for specialist care may be mitigated.

Our experience suggests that training of maternity nursing staff to screen and refer for mental health care makes use of existing resources to integrate mental health services, as well as building the capacity of health workers. Using a task-sharing approach, and with on-going supervision and support, the PMHP model delegates service responsibilities from higher to lower cadres of health staff, reducing the need for specialist mental health providers [24],[25]. Maternity staff trained by PMHP report improved capability to identify women's mental health problems and to assist women with these [26].

Established protocol and referral systems enable more effective case management. Where women need mental health services, the availability of an on-site counsellor allows staff to make a direct referral and ensure that there is continuity of care. Rather than adding a burden to their workload, staff reported a sense of relief that systems have been developed to meet the previously ignored need [26].

The high coverage (90%) and uptake (95%) of PMHP screening may be attributed to various factors, such as the consistency with which the health care staff offered the screening or the involvement of the clinical coordinator in motivating and supervising the staff to conduct screening. It may also be due to the investment in training of health care staff, which is regarded as an integral part of the PMHP stepped care model.

There appears to be demonstrated feasibility and acceptability of the counselling intervention, despite the relatively low number of sessions received (an average of 2.7 amongst this cohort). The preliminary evaluation of the counselling intervention appears strongly positive. This coheres with other evidence demonstrating that even one or two contact sessions are beneficial [27],[28]. However, the PMHP evaluation data were self-reported and not collected using standardised assessment measures. The project is currently undertaking a formal controlled evaluation of the counselling intervention to examine the efficacy of the counselling service.

## Transferability of the Model

In the South African obstetric system, postnatal care is limited, which severely impacts the PMHP's ability to conduct screening during this period. However, women who have already accessed the PMHP service antenatally may continue to receive counselling postnatally. In other settings, where maternal care is more comprehensive, and both antenatal and postnatal visits occur at the same site, the PMHP model may be adapted to include the detection of postnatal mental disorders.

In order to scale up mental health services as a part of routine maternal care in South Africa, the PMHP has extended its model utilising lay counsellors to two other community obstetric sites in Cape Town. The monitoring and evaluation data from these sites have generated several new health systems lessons that have the potential to inform health system re-organisation.

In South Africa, the availability of resources and the quality of health care varies quite substantially in rural and urban areas [27],[28]. As all of the current PMHP sites are situated in urban areas, there is a need for the PMHP to establish a rural site in order to evaluate the model in diverse settings where fewer resources and different challenges exist.

Although the combined use of the EPDS and RFA screening tools has proven to be workable in some settings, a single and shorter screening instrument may prove to be more useful given the number of tasks that staff are required to complete during routine antenatal care. There is a possibility in the near future for the PMHP to develop a shorter screening tool, designed to facilitate ease of use in busy settings with high patient volumes.

## Conclusion

The project provides a mental health service in a real-world obstetric setting where resources are scarce and patient volumes are high. The PMHP model takes into account pragmatic issues such as the capacity development of general health workers to provide primary mental health care. Secondly, the PMHP model optimises access to care for vulnerable patients. These principles may inform the development of services in similar primary health settings. See Box 1 for main lessons learnt.

**Box 1. Lessons Learnt**

1. Maternity health workers may be trained to screen and refer for mental distress in low-resource primary care settings.
2. Training programmes that address and support the mental health needs of health workers may help staff to manage their workload and prevent compassion fatigue and “burn out”.
3. On-site screening and counselling fosters the establishment of efficient referral mechanisms and access to mental health care often lacking in maternity settings in LMIC.
4. On-site, integrated mental health services increase access for women who have scarce resources and competing health, family, and economic priorities.
5. Coordinating mental health visits with subsequent antenatal visits further facilitates access for women with insufficient resources.
6. Dedicated, supervised mental health counselling personnel are required to meet the mental health needs of mothers living in adversity.
7. Mental health counsellors require adequate training, supervision, and support to manage the high case load generated in low-resource settings.

## Abbreviations

EPDS: Edinburgh Postnatal Depression Scale
HIC: high-income country
LMIC: low- and middle-income country
PMHP: Perinatal Mental Health Project
RFA: risk factor assessment

## References

[1] Mayosi BM, Flisher AJ, Lalloo UG, Sitas F, Tollman SM (2009). The burden of non-communicable diseases in South Africa.. Lancet.

[2] World Health Organisation Department of Health Statistics and Informatics (2008). The global burden of disease 2004 update.

[3] Fisher J, Mello CD, Patel V, Rahman A, Tran T (2012). Prevalence and determinants of common perinatal mental disorders in women in low- and lower-middle-income countries: a systematic review.. B World Health Organ.

[4] Walker SP, Wachs TD, Gardner JM, Lozoff B, Wasserman GA (2007). Child development: risk factors for adverse outcomes in developing countries.. Lancet.

[5] Oates M (2003). Perinatal psychiatric disorders: a leading cause of maternal morbidity and mortality.. Brit Med Bull.

[6] World Health Organisation (2008). Maternal mental health and child health and development in low and middle income countries: report of the meeting held in Geneva, Switzerland, 30 January–1 February, 2008.

[7] Rahman A, Lovel H, Bunn J, Iqbal Z, Harrington R (2003). Mothers mental health & infant growth: a case-control study from Rawalpindi, Pakistan.. Child Care Health Dev.

[8] Patel V, Rahman A, Jacob KS, Hughes M (2004). Effect of maternal mental health on infant growth in low income countries: new evidence from South Asia.. BMJ.

[9] Cooper PJ, Murray L, Wilson A, Romaniuk H (2003). Controlled trial of the short- and long-term effect of psychological treatment of post-partum depression.. Brit J Psychiat.

[10] Hartley M, Tomlinson M, Greco E, Comulada WS, Stewart J (2011). Depressed mood in pregnancy: prevalence and correlates in two Cape Town peri-urban settlements.. Reprod Health.

[11] Cooper PJ, Tomlinson M, Swartz L, Woolgar M, Murray L (1999). Post-partum depression and the mother-infant relationship in a South African peri-urban settlement.. Brit J Psychiat.

[12] Rochat TJ, Tomlinson M, Bärnighausen T, Newell M-L, Stein A (2011). The prevalence and clinical presentation of antenatal depression in rural South Africa.. J Affect Disord.

[13] Shisana O, Simbayi L, Rehle T, Zungu N, Zuma K (2008). South African national HIV prevalence, incidence, behaviour and communication survey. The health of our children.

[14] Openshaw MR, Bomela HN, Pretlove S (2011). An evaluation of the timing and use of healthcare during pregnancy in Birmingham, UK and Pretoria, South Africa.. ISRN Obstet Gynecol.

[15] Chopra M, Daviaud E, Pattinson R, Fonn S, Lawn JE (2009). Saving the lives of South Africa's mothers, babies, and children: can the health system deliver?. Lancet.

[16] Chopra M, Lawn JE, Sanders D, Barron P, Abdool Karim SS (2009). Achieving the health Millennium Development Goals for South Africa: challenges and priorities.. Lancet.

[17] Rojas G, Fritsch R, Solis J, Jadresic E, Castillo C (2007). Treatment of postnatal depression in low-income mothers in primary-care clinics in Santiago, Chile: a randomised controlled trial.. Lancet.

[18] Araya R, Rojas G, Fritsch R, Gaete J, Rojas M (2003). Treating depression in primary care in low-income women in Santiago, Chile: a randomised controlled trial.. Lancet.

[19] Cox J, Chapman G, Murray D, Jones P (1996). Validation of the Edinburgh postnatal depression scale (EPDS) in non-postnatal women.. J Affect Disord.

[20] Lawrie TA, Hofmeyr GJ, de Jager M, Berk M (1998). Validation of the Edinburgh Postnatal Depression Scale on a cohort of South African women.. S Afr Med J.

[21] Husain N, Bevc I, Husain M, Chaudhry IB, Atif N (2006). Prevalence and social correlates of postnatal depression in a low income country.. Archives of Women's Mental Health.

[22] Josefsson A, Angelsiöö L, Berg G, Ekström C-M, Gunnervik C, Nordin C (2002). Obstetric, somatic, and demographic risk factors for postpartum depressive symptoms.. Obst Gynecol.

[23] Austin M-P (2004). Antenatal screening and early intervention for “perinatal” distress, depression and anxiety: where to from here?. Arch Women Ment Health.

[24] Patel V, Weiss H, Chowdhary N, Naik S, Pednekar S, Chatterjee S (2010). Effectiveness of an intervention led by lay health counsellors for depressive and anxiety disorders in primary care in Goa, India (MANAS): a cluster randomised controlled trial.. Lancet.

[25] Zachariah R, Ford N, Philips M, Lynch S, Massaquoi M, Janssens V (2009). Task shifting in HIV/AIDS: opportunities, challenges and proposed actions for sub-Saharan Africa.. Trans R Soc Trop Med Hyg.

[26] Chesselet J (2005). The Perinatal Mental Health Project: a qualitative evaluation [master's dissertation].

[27] Coovadia H, Jewkes R, Barron P, Sanders D, McIntyre D (2009). The health and health system of South Africa: historical roots of current public health challenges.. Lancet.

[28] Lund C, Kleintjes S, Kakuma R, Flisher AJ (2010). Public sector mental health systems in South Africa: inter-provincial comparisons and policy implications.. Soc Psychiatry Psychiatr Epidemiol.

